# Non-typhoidal *Salmonella* among slaughterhouse workers and in the pork value chain in selected districts of Uganda

**DOI:** 10.3389/fvets.2024.1427773

**Published:** 2024-09-17

**Authors:** Velma Kivali, Kristina Roesel, Ian Dohoo, Lordrick Alinaitwe, James Katamba Bugeza, Jolly Justine Hoona, Denis Rwabiita Mugizi, Clovice Kankya, Sinh Dang-Xuan, Istvan Szabo, Uwe Rösler, Anika Friese, Elizabeth A. J. Cook

**Affiliations:** ^1^Animal and Human Health Program, International Livestock Research Institute, Kampala, Uganda; ^2^Animal and Human Health Program, International Livestock Research Institute, Nairobi, Kenya; ^3^Institute for Animal Hygiene and Environmental Health, Freie University of Berlin, Berlin, Germany; ^4^Department of Health Management, Atlantic Veterinary College, University of Prince Edward Island, Charlottetown, PE, Canada; ^5^Veterinary Public Health Institute, University of Bern, Bern, Switzerland; ^6^Graduate School for Cellular and Biomedical Sciences, University of Bern, Bern, Switzerland; ^7^Department of Animal Production, Ministry of Agriculture, Animal Industry and Fisheries, Entebbe, Uganda; ^8^College of Veterinary Medicine, Animal Resources and Biosecurity, Makerere University, Kampala, Uganda; ^9^Department for Biological Safety, German Federal Institute for Risk Assessment, BfR, Berlin, Germany

**Keywords:** non-typhoidal *Salmonella*, slaughterhouse workers, pork value chain, NTS serovars, Uganda

## Abstract

**Introduction:**

Non-typhoidal *Salmonella* (NTS) is a major cause of gastroenteritis worldwide, often associated with meat consumption and meat processing. Research on NTS infection and circulating serovars in meat value chains in Uganda is limited. We aimed to establish NTS prevalence, antimicrobial resistance, and risk factors among slaughterhouse workers, and to identify potentially zoonotic serovars in the pork value chain.

**Material and methods:**

We conducted a nationwide cross-sectional survey, collecting 364 stool samples from livestock slaughterhouse workers and 1,535 samples from the pork value chain: mesenteric lymph nodes, fecal samples, swabs of carcass splitting floor, cleaning water, meat handlers hand swabs, carcass swabs, raw pork, cooked pork, and mixed raw vegetables. Samples were cultured for isolation of NTS, and subsequently serotyped according to White–Kauffmann–Le Minor scheme. Antimicrobial resistance profiles were determined using tube microdilution and Sensititre^®^ EUVSEC3^®^ plates. Semi- structured questionnaires with 35 questions were used to collect data on demographics, work related risk factors and activities outside the slaughterhouse.

**Results and discussion:**

Overall NTS prevalence was 19.2% (365/1899). Proportions at slaughter were; 46.7% in floor swabs, 30.5% in carcass swabs, 20.5% in pig faeces,19.2% in mesenteric lymph nodes,18.4% in hand swabs, 9.5% in water and 5.2% in slaughterhouse workers. At retail, proportions were 33.8% in pork chopping surface, 33.1% in raw pork, 18.9% in hand swabs, 4.0% in cooked pork and 0.7% in vegetables. Sixty-one serovars were identified, with significant overlap between humans and the pork value chain. Overall, zoonotic *S*. Zanzibar, monophasic serovars of *S*. subspecies *salamae* (II) and subspecies *enterica* (I), *S*. Typhimurium and *S*. Newport, were the most prevalent. *S*. Typhimurium was predominant in humans and exhibited multi-drug resistance. NTS infection was significantly associated with eating, drinking, or smoking while working (OR = 1.95, 95% CI: 0.67-2.90%, *p* = 0.004). The detected NTS serovars in slaughterhouse workers could be a potential indicator of circulating serovars in the general population. The persistent presence of NTS along the pork value chain highlights occurrence of cross-contamination and the potential for transmission to consumers and slaughterhouse workers. This emphasizes the need to reduce *Salmonella* prevalence on pig farms and improve hygiene and pork handling practices at slaughter and retail points.

## Introduction

Unsafe foods and foodborne diseases (FBD) are a global public health concern, causing millions of infections and more than 420,000 deaths annually (1). The most common FBD are diarrheal illnesses, which cause 550 million infections each year, including 220 million cases in children under the age of five (2). Non-typhoidal *Salmonella* (NTS) infection is among the leading causes of diarrheal infections, and it is estimated to cause 93.8 million enteric infections and 155,000 diarrheal deaths worldwide annually (2, 3).

In the European Union, NTS was the second most commonly reported zoonotic agent of food-borne diseases after *Campylobacter* spp., and the leading cause of foodborne illnesses in 2022 (4). Similarly, in the United States, NTS ranks second after norovirus among the leading causes of foodborne illnesses, but it is the leading cause of hospitalizations and deaths related to food borne diseases (5). In the sub-Saharan Africa region, NTS remains a key source of food contamination, associated with high morbidity and mortality, and accounting for 32,000 annual deaths (6).

NTS infections are caused by salmonellae, which *are* gram-negative, rod-shaped intracellular bacteria in the family Enterobacteriaceae. They belong to the genus *Salmonella* which comprises two main species: *Salmonella bongori* and *Salmonella enterica* (7). Typically, NTS infections in humans and animals are caused by serovars of *S. enterica* except for Typhi, Paratyphi A, Paratyphi B, and Paratyphi C (8). The most common and epidemiologically important serovars associated with causing human outbreaks worldwide are Typhimurium, Enteritidis, Heidelberg, and Newport (9).

Transmission of NTS to humans occurs through ingestion of contaminated water and food, contact with infected animals and through human-to-human transmission (2). Food animals, particularly pigs, are known to be a reservoir for NTS and an important source of transmission to humans (2, 10). Pigs are asymptomatic carriers, intermittently shedding the bacteria in feces, consequently contaminating pork and posing an occupational risk to meat handlers in the pork value chain (11).

At the point of slaughter, sources of NTS include animal feces, animal hides, and contaminated slaughter environments (12, 13). The risk of exposure to NTS among slaughterhouse workers is largely determined by hygiene and meat handling practices (14). Additional factors influencing exposure levels include the specific role of the meat handler within the chain and the frequency of interaction with slaughtered animals, meat, and the slaughter environment (15). There is evidence of transmission of NTS from pigs to humans through the pork value chain, observed through serovar analysis across various matrices and foodborne outbreaks linked to pork consumption (10).

In humans, NTS infection typically causes gastroenteritis, which is self-limiting in otherwise healthy individuals (16). However, in severe cases, especially among high-risk populations such as children, the elderly, and immunocompromised individuals, treatment with antibiotics, mainly fluoroquinolones and third-generation cephalosporins is required (17). Additionally, non-typhoidal salmonellae are associated with the invasive form of the disease, known as invasive non-typhoidal *Salmonella* (iNTS), which is endemic in sub-Saharan Africa (18). iNTS is characterized by bacteraemia, mostly in the absence of diarrhea and has a case fatality rate of 20–25% in sub-Saharan Africa, compared to 1–5% in high income countries (19, 20). The key risk factors are HIV infection in adults, and malaria, HIV, and malnutrition in children (19). The main serovars associated with iNTS are *S*. Typhimurium sequence type (ST) 313 and *S*. Enteritidis (18).

Globally, antimicrobial resistance (AMR) associated with NTS is on the rise. This increase is largely attributed to the widespread use of antimicrobials in food animals, which leads to the transfer of resistant *Salmonella enterica* to humans through the food chain (17, 21). The development of multi drug resistance has been widely reported in various *S*. enterica serovars in humans, and it is on the rise in the African region (21).

In high-income countries, there is continuous active or passive monitoring of NTS infections and antimicrobial resistance, and integrated harmonized control programs (22). However, in low- and middle-income countries, there is absence of surveillance, monitoring and reporting hence the burden of NTS remains unknown (23). Crump et al. (22) reported that meat pathways are an important potential source of transmission of some clades of *S*. Enteritidis to humans in East Africa, suggesting the need for more research. Surveillance at slaughter houses can help in establishing NTS and AMR monitoring in both animals and humans at the same time (24).

In Uganda, like in most low- and middle-income countries, the burden of NTS in the different meat value chains and in humans, particularly slaughterhouse workers remains unknown. Studies done have investigated *Salmonella* in archived NTS human isolates from clinical cases and in patients in hospital settings (25–27). Afema et al. (25) reports that NTS from humans, livestock and environmental sources had shared genotypes and AMR phenotypes, suggesting a possibility of zoonotic transmission of NTS. There is also evidence of cross-species transmission of plasmids, and possibly drug resistance, between food animals and humans, emphasizing the role played by food animals in transmission of NTS (26). Kagirita et al. (27) demonstrates that there are shared serovars between animals and humans in Uganda.

Our study therefore aimed to establish the prevalence of NTS in slaughterhouse workers, risk factors associated with infection, phenotypic antimicrobial-resistance profiles, and prevalence of NTS along the pork value chain from slaughter to retail, while identifying potentially zoonotic NTS serovars.

## Methods

### Ethics statement

Ethical approval for this research was reviewed and approved by the Makerere School of Public Health (MAKSHSREC-2021-109), Makerere School of Biosecurity, Biotechnology and Laboratory Sciences (SBLS/HDRC/20/014), and the Uganda National Council for Science and Technology (Research registration number HS1820ES). Written consent was sought from each of the individual study participants, before questionnaire administration and sample collection. The consent forms were translated to local dialects with the help of native speakers and in cases where a participant could not read and write, the consent was read to them and they would sign by thumb printing. To ensure anonymity, each participant was given a unique coded identifier, that was used for their samples and the metadata.

### Study design and area

**Figure 1:**
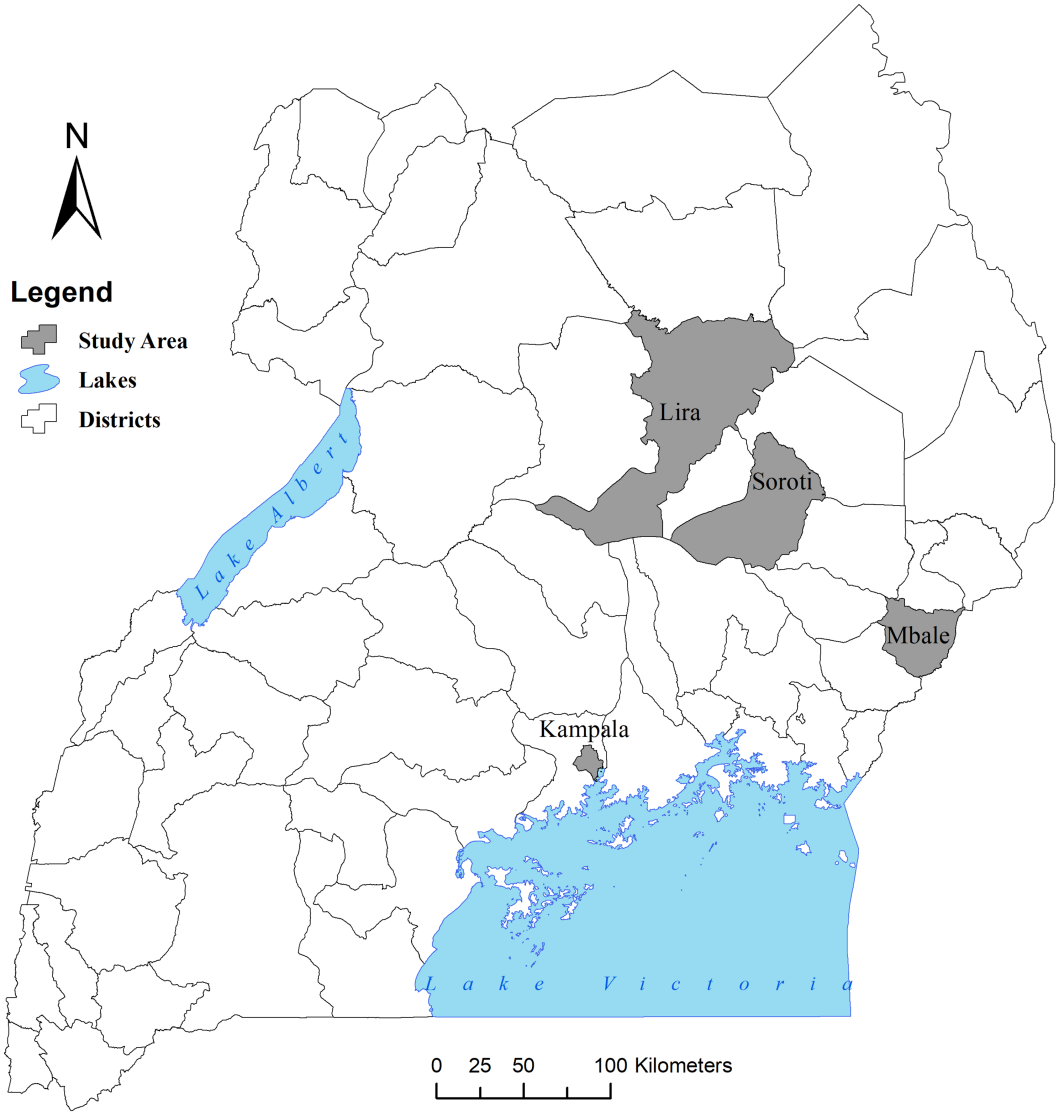
Map of Uganda showing the districts of the study. Source: Stephen Oloo/ILRI. Map drawn in open QGIS version 3.24.2 with a base layer of Uganda districts downloaded from the Humanitarian Data Exchange database (https://data.humdata.org/).

Our cross-sectional study was conducted in ruminant and pig slaughter facilities in three regions of Uganda between December 2021 and December 2022. Four districts were purposively selected to represent the respective regions: Kampala in central region, Mbale and Soroti, in the eastern region and Lira in the northern region (Figure 1). Slaughterhouse workers from both ruminant and pig slaughter facilities were included in the study. However, sampling within the value chain was specifically focused only on pig slaughterhouses and pork retail points.

### Sample size determination

EPITOOLS online sample size calculator was used to estimate the sample size for slaughterhouse personnel (28). Using an assumed prevalence of 0.18, the slaughterhouse worker’s sample size was determined to be 227 (29).

The pork value chains differed between the central region and the other regions. In the central region pigs were slaughtered in one main slaughterhouse and distributed to multiple retail points, whereas in the other regions pigs were slaughtered in multiple slaughterhouses and distributed to one or more than one retail points. For this reason, different sample size calculations were conducted for each value chain.

The sample sizes for the pork value chain were determined in STATA, using the prevalence of *Salmonella* (10%) as an indicator pathogen for microbial contamination (30). For the central region, sample size calculation was performed assuming a comparison of paired means, i.e., following the same pig and its carcass starting from when a pig is slaughtered, through the slaughter process up to the point of retail. Using an assumed baseline prevalence of *Salmonella* of 10%, a standard deviation of 0.32 and assuming a correlation of 0.2 between slaughter and retail points and 80% power to detect a 15% change in prevalence of *Salmonella* between different points along the chain. This gave a target sample size of 60 pigs at the point of slaughter.

For the northern and eastern regions, the number of pork retail points to be included in the study was determined assuming a baseline prevalence of *Salmonella* of 10%, an average of 3 pigs per point of pork retail, a standard deviation between pork joint means of 0.19 and a correlation of 0.2 between slaughter and retail samples and an 80% power to detect a difference of 15% in the mean prevalence of *Salmonella* between different points along the chain, giving a total target of 69 pigs from 23 pork retail points.

### Sampling slaughterhouse workers

We organized a medical camp in each of the study areas during which stool samples were collected from consenting workers. The workers were mobilized and invited to attend the medical camp at will and at their convenience. Participants were recruited conveniently into the study, and only those who consented were sampled (Supplementary Data Sheet 1). Individuals who were not directly involved in the slaughter process, those who were below 18 years and those who did not consent were excluded. Materials and instructions for stool collection were delivered by trained nurses. At the same time, a digital pre-tested questionnaire (Supplementary Data Sheet 2), developed in Open Data Kit (31) was administered by trained interviewers both in English and in pre-translated local dialects. The questionnaire considered information on the respondents’ demographics (age, sex, religion, and education), work related risky behaviors (eating, drinking, and smoking while working), potential risk factors to *NTS* exposure, activities outside work and recent self-reported medical illness. A total of 364 human stool samples were collected. The collected stool samples were transported at 4°C in cooler boxes with ice packs to the Central Diagnostics Laboratories (CDL) at Makerere University, Kampala, on the same day for analysis, except during sampling in the eastern and northern regions where delivery was the next day. In this case, the samples were maintained at 4°C and shipped overnight in cooler boxes with ice packs to arrive in Kampala the next morning.

### Sampling at pig slaughter and pork retail points

In collecting samples for the pork value chain, 16 pig slaughter facilities and 62 points of pork retail were recruited across the three regions of Uganda. We created a sampling schedule based on the points of retail, detailing from which slaughter slab each retail point sourced their pork. We aimed at making two collection rounds for each retail point in the central region, and three collection rounds at each pork retail point in the northern and eastern regions. In central region we sampled 70 pigs at one slaughterhouse and followed the carcasses to the retail outlets (*n* = 35) for further sampling. In the northern region we sampled pigs from 6 slaughter facilities and 15 retail points, and in the eastern region we sampled pigs from 9 slaughter facilities and 12 retail points. Each facility (*n* = 27) was visited three times, sampling a total of 81 pigs.

At slaughter, the following samples were collected: approximately 30 g of mesenteric lymph nodes (MLN), made up of at least 5 nodes, about 30 g of pig fecal material from an incision made in the large intestines, a swab of both hands of the main meat handler, 200 cm^2^ swab of the carcass splitting floor, 200 mL water used for cleaning carcasses, the main source was municipal tap water but was often used from secondary containers, and 400 cm^2^ swab (ham, back, belly, and jowl) of each of the carcasses as described by the International Organization for Standardization (ISO) in ISO-17604, 2015 (32). Demographic details that included age (adult/juvenile), sex, breed, and the district of origin of the slaughtered pigs were also collected. Upon their dispatch from the slaughterhouse, two field assistants followed the selected carcasses to the retail destination where additional samples were picked within 1–4 h as follows: 50 g of the raw pork, a swab of both hands of the pork handler, a 200 cm^2^ swab of the chopping surface, 50 g of cooked pork, and 50 g of mixed raw vegetables commonly referred to as kachumbari (finely chopped and mixed raw onions, tomatoes, and coriander). A total of 1,535 samples were collected from slaughter and retail points of the pork value chain: 151 mesenteric lymph node samples, 151 pig fecal samples, 151 carcass swabs, 295 hand swabs of the meat handlers, 137 water samples, 137 swabs of the carcass splitting floor, 151 swabs of the pork chopping surface at retail, 151 raw pork samples, 151 cooked pork samples and 60 mixed raw vegetable samples. The samples were delivered to CDL for analysis as described above.

### Culture and isolation of *Salmonella*

Detection of *Salmonella* was carried out according to ISO 6579-1:2017 (33) in four different stages: pre-enrichment, selective enrichment, plating out, and identification. Briefly, the samples were homogenized in a ratio of 1:10 in buffered peptone water (Oxoid^™^ Hampshire, England) and incubated at 37°C for 24 h. Subsequently, 1 and 0.1 mL of the pre-enriched homogenates were transferred into 9 mL Muller–Kauffmann tetrathionate novobiocin broth (Oxoid^™^, Hampshire, England) and 10 mL of Rappaport-Vassiliadis soy peptone broth (Oxoid^™^, Hampshire, England), and incubated at 37°C for 24 h, and at 42°C for 24 h, respectively. A 10 μL loop from each broth was streaked onto Xylose lysine tergitol agar-4 (XLT-4, Oxoid^™^ Hampshire, England) and *Salmonella* chromogenic agar base (Oxoid^™^ Hampshire, England) with *Salmonella* selective supplement (Oxoid^™^ Hampshire, England) and incubated at 37°C for 24 h. Morphological colony characteristics of *Salmonella*, i.e., black colonies with yellow or pink periphery on XLT4 and magenta colonies on *Salmonella* chromogenic agar were examined. Biochemical tests [citrate −ve, methyl red +ve, urease −ve, hydrogen sulfide +ve, indole −ve, motility +ve, lactose −ve, dulcitol −ve, and sucrose −ve] were carried out and all the presumptive isolates stocked in brain heart infusion broth with 20% glycerol and stored at −20°C until needed for further isolate characterization. During microbial analysis in the laboratory, we ensured proper labeling, correct incubation temperatures and time, avoided cross-contamination by changing tips and followed the ISO protocol.

### Serotyping of *Salmonella* isolates

The presumptive NTS isolates were shipped on nutrient agar slants (Oxoid^™^, Hampshire, England) at room temperature to the Institute for Animal Hygiene and Environmental Health, Freie University of Berlin, Berlin, Germany. This was done in compliance with the Uganda National Council of Science and Technology regulations on Access and Benefits Sharing (A139ES). The isolates were first revived on Xylose Lysine Deoxycholate (Merck, Germany) agar plates and sub-cultured onto Colombia Blood agar plates (Thermo Scientific^™^, UK). Single pure colonies from the blood agar plates were then serotyped using anti-Salmonella A-67 + Vi, Omnivalent (Sifin diagnostics, Germany) and additionally confirmed to be *Salmonella* using the Matrix-Assisted Laser Desorption/Ionization Time-of-Flight (MALDI-TOF MS). Confirmatory serotyping was done at the German Federal Institute of Risk Assessment, Berlin, Germany. Single colonies from pure cultures were serotyped using the O and H antisera (Bio-Rad, Germany) according to ISO/TR 6579–3 and classified according to the White–Kauffmann–Le Minor scheme (7). Isolates showing a rough phenotype in slide agglutination, were subjected to a *Salmonella* specific polymerase chain reaction (PCR), to confirm the genus (34).

### Antimicrobial susceptibility testing

Only isolates from humans were tested for resistance against selected antibiotics using the minimum inhibition concentration technique. The test panel comprised 11 antibiotics preloaded in microtiter plates (Sensititre^®^ EUVSEC3^®^ plates, Thermofisher Scientific, Paisley, UK). Briefly, 1–5 colonies were put in sterile physiological saline solution to obtain a homogeneous solution of 0.5 McFarland. A volume of 60 μL of the suspension was added into 11 mL cation adjusted Muller-Hinton broth to achieve a final titer of approximately 5 × 10^−5^ CFU/mL. Subsequently, a volume of 50 μL was dispensed per well into EUVSEC3^®^ plates. The plates were sealed and incubated at 35°C for 18 h in a non-CO_2_ incubator. The following day, the minimum inhibition concentration in μg/ml was determined. The antibiotics on the EUVSEC3^®^ plate were ampicillin (1–32 μg/mL), chloramphenicol (8–64 μg/mL), ciprofloxacin (0.015–8 μg/mL), colistin (1–16 μg/mL), gentamicin (0.5–16 μg/mL), nalidixic acid (4–64 μg/mL), sulfamethoxazole (8–512 μg/mL), tetracycline (2–32 μg/mL), trimethoprim (0.25–16 μg/mL), azithromycin (2–64 μg/mL), and tigecycline (0.25–8 μg/mL). The European Committee on Antimicrobial Susceptibility (EUCAST) thresholds for resistance and concentration ranges for *Salmonella* spp. were adopted for the interpretation of the resistance patterns (35).

### Data management and statistical analysis

All the data was stored in Microsoft Excel (Microsoft 365, Version 2111). Descriptive statistics were used to describe the detection prevalence of *Salmonella enterica* serovars. Statistical analyses were carried out in R version 4.2.3 (36). Univariable logistic regression was conducted using the *glm* function in R to identify variables with a *p*-value less than 0.05. Subsequently, a multivariable logistic regression model was developed using backward stepwise elimination, starting with all variables identified in the univariable analyses. Only variables with a *p*-value of 0.05 or less were retained in the final model.

## Results

### Prevalence of non-typhoidal *Salmonella*

Overall NTS prevalence was 19.2% (365/1899; 95% CI: 17.5–21.0) (Table 1). Prevalence in livestock slaughterhouse workers was 5.2% (19/364; 95% CI: 3.4–8.0), 23.9% (209/874; 95% CI: 21.2–26.8) in samples collected at pig slaughter; and 20.7% (137/661; 95% CI: 17.7–24.0) in samples from pork retail. Slaughtered pigs had a prevalence of 19.2% (29/151; 95% CI: 13.8–26.1) in MLN and 20.5% (31/151; 95% CI: 14.8–27.7) in fecal samples. Floor swabs had the highest NTS prevalence 46.7% (64/137; 95% CI: 38.5–54.9) and vegetables the lowest prevalence 0.7% (1/60; 95% CI: 0.2–3.6). Chopping surfaces had the highest prevalence at retail 33.8% (51/151; 95% CI: 26.8–41.5). Prevalence in carcasses leaving the point of slaughter was 30.5% (46/151; 95% CI: 23.7–38.1), in raw pork arriving at the point of retail, 33.1% (50/151; 95% CI: 26.2–40.7) and 4.0% (6/151; 95% CI: 1.8–8.4) in cooked ready to eat pork.

**Table 1:** Non-typhoidal Salmonella in livestock slaughterhouse workers, and in the pork value chain, from Lira, Mbale, Soroti and Kampala districts of Uganda.

Samples types	No. of *Salmonella* positive/No. of samples (%)			
	Slaughterhouse		Pork retail	Overall
	Ruminant	Pig		
Human				
Stool	11/242 (4.5)	8/122 (6.6)		19/364 (5.2)
Hands swab		27/147 (18.4)	28/148 (18.9)	55/295 (18.6)
Environment				
Floor swab		64/137 (46.7)		64/137 (46.7)
Water for cleaning carcass		13/137 (9.5)		13/137 (9.5)
Chopping surface			51/151 (33.8)	51/151 (33.8)
Animal (pig)				
Mesenteric lymph node		29/151 (19.2)		29/151 (19.2)
Fecal		31/151 (20.5)		31/151 (20.5)
Carcass swab		46/151 (30.5)		46/151 (30.5)
Raw pork			50/151 (33.1)	50/151 (33.1)
Cooked pork			6/151 (4.0)	6/151 (4.0)
Raw vegetables			1/60 (0.7)	1/60 (0.7)
Subtotals	11/242 (4.5)	218/996 (21.9)	136/661 (20.6)	365/1899 (19.2)

No., number: %, percentage.

### Non-typhoidal *Salmonella* serovar distribution in stool samples from slaughterhouse workers

In stool samples collected from workers, serotyping confirmed 11 different serovars from the 19 isolates: *S*. Typhimurium was the most common serovar in eight samples, followed by two *S.* Zanzibar and one of each of the other serovars (Table 2). All the *S*. Typhimurium isolates were from slaughterhouse workers in a ruminant slaughter facility in Mbale, eastern region. Out of the 11 serovars, 8 were also detected in the pork value chain, while *S*. Kenya, *S*. Moroto and *S*. Sanjuan were found only in humans. All the isolates tested for MIC were sensitive to ampicillin, chloramphenicol, ciprofloxacin, colistin, gentamicin, nalidixic acid, trimethoprim, and azithromycin. Seven out of the nineteen (36.8%) confirmed serovars tested for MIC showed multiple-drug resistance to commonly used antimicrobials, i.e., sulfamethoxazole, tetracycline, and tigecycline. Resistance was only detected in the *S*. Typhimurium serovars. Additionally, 9/19 (47.4%) of those with NTS had at least one of the clinical symptoms associated with NTS infection, i.e., diarrhea, vomiting, headache, fever, nausea, or abdominal pain.

**Table 2:** Non-typhoidal *Salmonella* serovars and antimicrobial resistance profiles from slaughterhouse workers (stool samples) from Lira, Mbale, Soroti and Kampala districts, Uganda between 12/2021 and 12/2022.

No	Slaughterhouse type	Region	Serovar	Clinical symptom[s]*	Antimicrobial resistance profile
1	Ruminant	Northern	*S.* Sanjuan	Asymptomatic	Sensitive
2	Ruminant	Northern	*S. enterica* subspecies *salamae*, 42:r:-	Symptomatic	Sensitive
3	Pig	Northern	*S.* Kenya	Symptomatic	Sensitive
4	Pig	Northern	*S.* Ituri	Asymptomatic	Sensitive
5	Pig	Northern	*S.* Moroto	Symptomatic	Sensitive
6	Pig	Northern	*S.* Zanzibar	Symptomatic	Sensitive
7	Pig	Northern	*S.* Offa	Symptomatic	Sensitive
8	Ruminant	Eastern	*S*. Typhimurium	Asymptomatic	SMX, TET, TGC
9	Ruminant	Eastern	*S*. Typhimurium	Symptomatic	SMX, TET, TGC
10	Ruminant	Eastern	*S*. Typhimurium	Asymptomatic	SMX, TET, TGC
11	Ruminant	Eastern	*S*. Typhimurium	Symptomatic	SMX, TET, TGC
12	Ruminant	Eastern	*S*. Typhimurium	Asymptomatic	SMX, TET, TGC
13	Ruminant	Eastern	*S*. Typhimurium	Asymptomatic	SMX, TET, TGC
14	Ruminant	Eastern	*S*. Typhimurium	Symptomatic	SMX, TET, TGC
15	Ruminant	Eastern	*S*. Typhimurium	Asymptomatic	Sensitive
16	Pig	Eastern	*S.* Adelaide	Asymptomatic	Sensitive
17	Ruminant	Central	*S.* Zanzibar	Symptomatic	Sensitive
18	Pig	Central	*S.* Newport	Asymptomatic	Sensitive
19	Pig	Central	*S.* Stanleyville	Asymptomatic	Sensitive

*Self-reported by workers during the interview on the day of sampling: SMX, sulfamethoxazole; TET, tetracycline; TGC, tigecycline.

### Non-typhoidal *Salmonella* serovar distribution in the pork value chain

**Figure 2:**
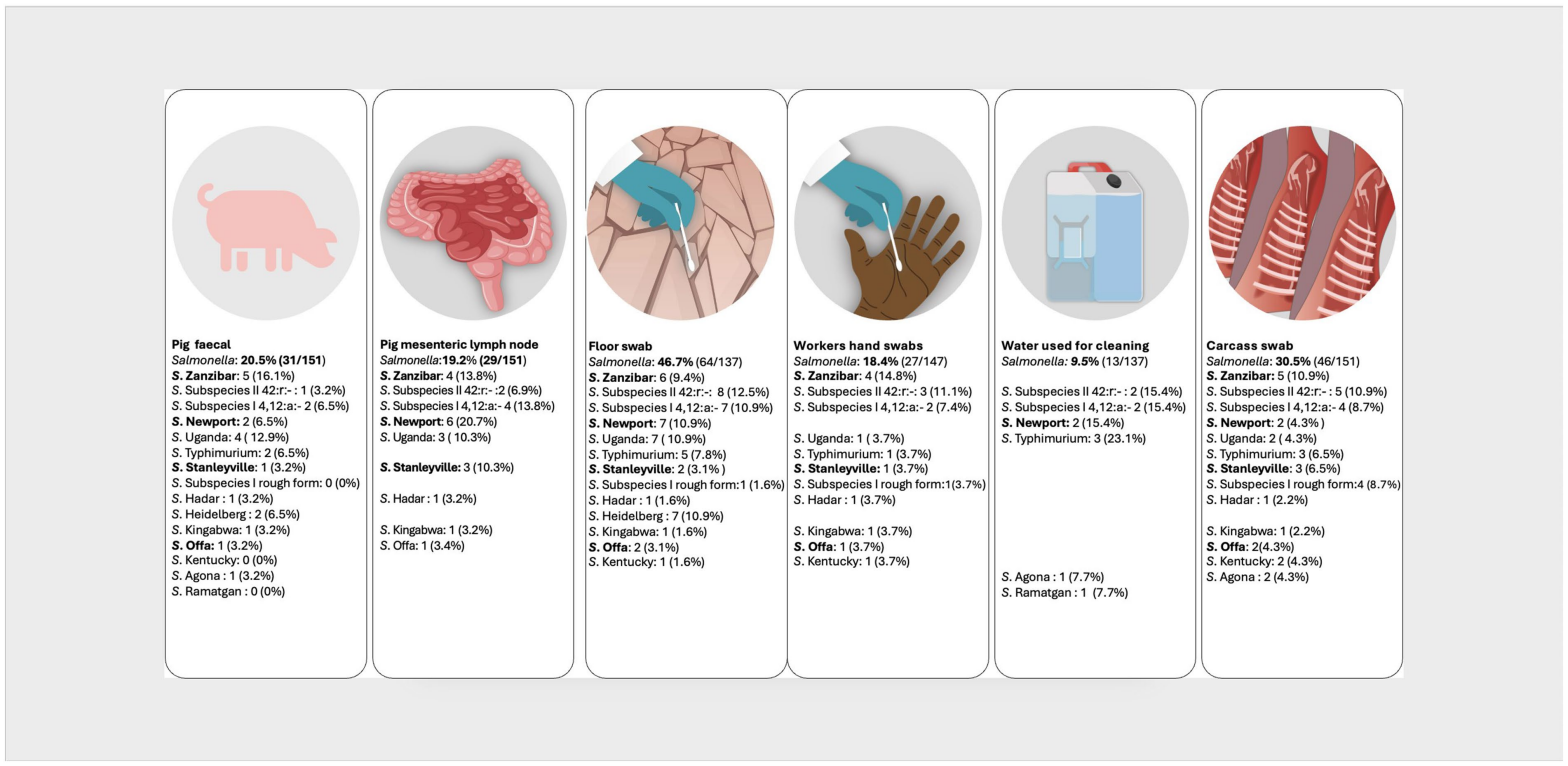
Distribution of the top 15 most prevalent serovars detected at the point of pig slaughter. Illustration: ILRI/Annabel Slater. (*Salmonella* prevalence at the top is the prevalence per sample type, while the prevalences next to the serovars shows how many of the positives belong to a particular serovar. Serovars in bold were also detected in pig slaughterhouse workers).

**Figure 3:**
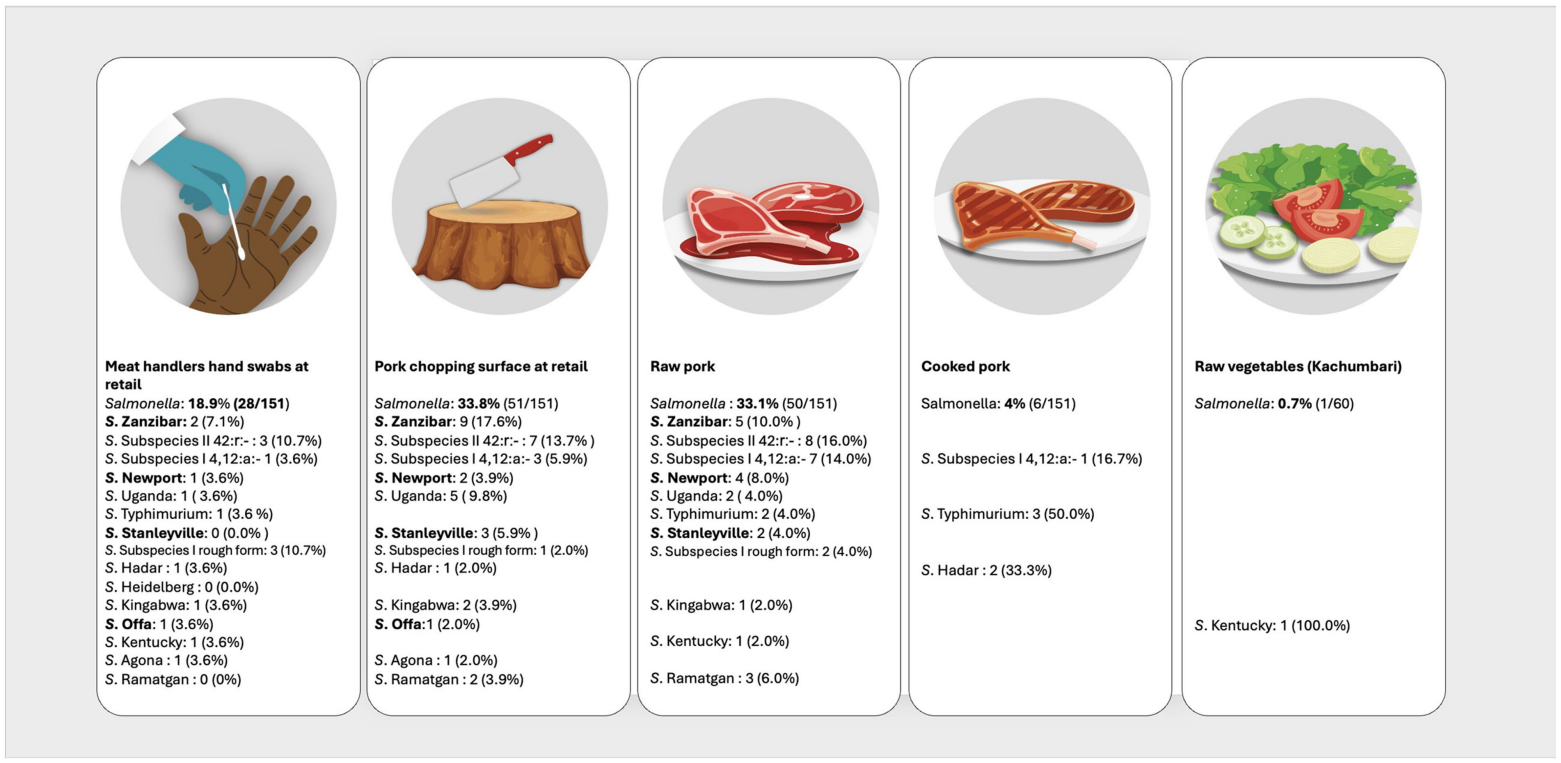
Distribution of the top 15 most prevalent serovars detected at the point of pork retail. Illustration: ILRI/Annabel Slater. (*Salmonella* prevalence at the top is the prevalence per sample type, while the prevalences next to the serovars shows how many of the positives belong to a particular serovar. Serovars in bold were also detected in pig slaughterhouse workers).

In the pork value chain, 58 different NTS serovars, characterized by unique antigenic formulas were identified in the different sample types (Supplementary Table 1). The fifteen most prevalent serovars were *S.* Zanzibar (11.6%, 40/346); *S. enterica* subspecies *salamae*, 42:r.- (11.3%, 39/346); monophasic *S*. subspecies *enterica*, 4, 12:a:- (9.5%, 33/346); *S*. Newport (7.5%, 26/346); *S.* Uganda (7.2%,25/346); *S*. Typhimurium (5.8%, 20/346); *S*. Stanleyville (4.3%, 15/346); *S.* subspecies *enterica* rough form (3.5%, 12/346); *S*. Hadar, *S*. Heidelberg, *S*. Kingabwa each at 2.6% (9/346); *S*. Offa (2.3%. 8/346); S. Kentucky (2.0%, 7/346); and *S*. Agona and *S*. Ramatgan each at (1.7%, 6/346). Figures 2, 3 show the 15 most common serovars detected at different points in slaughter and retail, respectively. We detected the same serovars at both the slaughter and retail for the same pig carcasses in 40/151 (26.4%) instances with the most common serovars detected at both slaughter and retail for the same carcass being *S. enterica* subspecies *salamae,* 42:r (*n* = 8), S. Zanzibar (*n* = 7), monophasic *S*. subspecies *enterica*, 4, 12:a:- (*n* = 6), *S*. Typhimurium (*n* = 4) and S. Uganda (*n* = 3) (Supplementary Table 2).

The other serovars included monophasic variations of serovars of *S. s*ubspecies *enterica* (9), variations of serovars of S. subspecies *enterica* (4), *S*. Enteritidis (5), *S*. Guildford (5), *S*. Adelaide (4), *S*. Teshi (4), *S.* Chicago (3), *S.* Hvittingfoss (3), *S.* Mikawasima (3), *S.* Nessziona (3), *S.* Os (3), 42:z39 (2), *S.* Aberdeen (2), *S.* Blijdorp (2), *S.* Cerro (2), *S.* Hull (2), *S.* Irchel (2), *S. Orion* (2), *S. Urbana* (2), *S*. subspecies *salamae*, 47:z:z6 (1), *S.* Amsterdam (1), *S.* Bolton (1), *S.* Bonn (1), *S.* Bovismorbificans (1), *S.* Braenderup (1), *S.* Eppendorf (1), *S.* Galiema (1), *S.* Ilala (1), *S.* Ituri (1), *S.* Kingston (1), *S.* Luke (1), *S.* Montevideo (1), *S.* Senftenberg (1), *S.* subspecies *salamae*, rough form (1), *S.* Tilene (1), *S.* Umbilo (1) and *S.* Zega (1).

Additionally, two isolates could not be serotyped, so they were confirmed as *Salmonella* by polymerase chain reaction. This was likely due to the breakage of the lipopolysaccharide layer, resulting in the loss of the specific O-group.

### Demographics of slaughterhouse workers

The 364 slaughterhouse workers were from 12 different cattle, pig, and small ruminant slaughter facilities across the areas of study. Three slaughterhouses were located in the northern region (two ruminant and one pig slaughter facility), 7 in the eastern region (two ruminant and 5 small pig slaughter slabs), and 2 in Kampala (one main ruminant and one main pig slaughter facility). Overall, 129 workers handled cattle only, 122 handled pigs only, 52 handled sheep and goats, 56 handled mixed ruminants (cattle, sheep and goats), and 5 worked with ruminants and pigs. The age ranged from 18 to 68 years with a mean age of 35 years. The duration worked at the slaughterhouse ranged between 1 month to 40 years with a mean of 9 years. Only 9.6% of the slaughterhouse workers reported having received training in at least one of the following thematic areas within 6 months before our data collection: meat hygiene, zoonotic diseases, occupational safety, and personal hygiene (Table 3).

**Table 3:** Results of univariable analysis for risk factors for non-typhoidal Salmonella infection in the slaughterhouse workers from Lira, Mbale, Soroti and Kampala districts, Uganda.

Variable	Number sampled (% of *n*)	*NTS* positive (%)	Odds ratio	95% CI	*p*-value
Region					
Northern	181 (49.4)	7 (3.9)	Ref	Ref	Ref
Eastern	89 (24.5)	9 (10.1)	2.80	1.01–8.08	0.049*
Central	94 (25.8)	3 (3.2)	0.82	0.17–3.02	0.777
Sex					
Female	40 (11.0)	2 (5.0)	Ref	Ref	Ref
Male	324 (89.0)	17 (5.2)	1.05	0.28–6.80	0.947
Age					
18–30 years	136 (37.4)	6 (4.4)	Ref	Ref	Ref
31–50 years	187 (51.4)	10 (5.3)	1.22	0.44–3.68	0.702
51–68 years	41 (11.3)	3 (7.3)	1.71	0.35–6.81	0.463
Education					
No formal education	21 (5.8)	1 (4.8)	Ref	Ref	Ref
Primary level	193 (53.0)	11 (5.7)	1.21	0.22–22.66	0.859
Secondary level	125 (34.3)	5 (4.0)	0.83	0.13–16.41	0.871
Tertiary level	25 (6.9)	2 (8.0)	1.74	0.16–39.09	0.661
Religion					
Christian	286 (78.6)	11 (3.8)	Ref	Ref	Ref
Muslim	78 (21.4)	8 (10.3)	2.86	1.07–7.33	0.029*
Training					
Yes	35 (9.6)	1 (2.9)	Ref	Ref	Ref
No	329 (90.4)	18 (5.5)	0.51	0.03–2.58	0.516
Years worked					
<1 year	27 (7.4)	1 (3.7)	Ref	Ref	Ref
1–5 years	145 (39.8)	6 (4.1)	1.39	0.23–26.49	0.761
6–10 years	81 (22.3)	6 (7.4)	2.28	0.36–43.98	0.455
>10 years	111 (30.5)	6 (5.4)	1.68	0.27–34.42	0.636
Role in the value chain					
Dirty role	101 (27.1)	5 (5.0)	Ref	Ref	Ref
Clean role	148 (38.2)	8 (5.4)	1.09	0.36–3.72	0.874
Both	115 (30.2)	6 (5.1)	1.06	0.31–3.77	0.929
Risky behaviors (eating/drinking/smoking)					
No	156 (42.9)	3 (1.9)	Ref	Ref	Ref
Yes	208 (57.1)	16 (7.7)	4.25	1.38–18.51	0.023*
Use of PPE (apron/gloves/gumboots/hair cover)					
No	75 (20.6)	4 (5.3)	Ref	Ref	Ref
Yes	289 (79.4)	15 (5.9)	0.80	0.31–2.61	0.724
Animal species					
Cattle only	129 (35.4)	4 (3.1)	Ref	Ref	Ref
Pigs only	122 (33.5)	8 (6.6)	2.19	0.67–8.40	0.210
Mixed species	113 (31.0)	7 (6.2)	2.06	0.61–8.06	0.258
Visible cuts on legs/hands					
No	151 (41.5)	7 (4.6)	Ref	Ref	Ref
Yes	213 (58.5)	12 (5.6)	1.23	0.48–3.37	0.674
Working with open wound					
No	110 (30.2)	4 (3.6)	Ref	Ref	Ref
Yes	264 (69.8)	15 (5.7)	1.66	0.59–5.94	0.376
Hand washing practices					
Before handling carcass					
No	207 (56.9)	12 (5.8)	Ref	Ref	Ref
Yes	157 (43.1)	7 (4.5)	0.76	0.28–1.93	0.571
After handling carcass					
No	111 (30.5)	8 (7.2)	Ref	Ref	Ref
Yes	253 (69.5)	11 (4.3)	0.59	0.23–1.55	0.264
Before eating					
No	268 (73.6)	13 (4.9)	Ref	Ref	Ref
Yes	96 (26.4)	6 (6.3)	1.31	0.45–3.42	0.598
After the toilet					
No	302 (83.0)	16 (5.3)	Ref	Ref	Ref
Yes	62 (17.0)	3 (4.8)	0.91	0.21–2.84	0.882
Following an injury					
No	344 (94.5)	18 (5.2)	Ref	Ref	Ref
Yes	20 (5.5)	1 (5.0)	0.95	0.05–5.02	0.964
Pre-existing medical conditions					
No	291 (79.9)	14 (4.8)	Ref	Ref	Ref
Yes	73 (20.1)	5 (6.8)	2.06	0.71–5.37	0.152
Keeping animals at home					
No	157 (43.1)	7 (4.5)	Ref	Ref	Ref
Yes	207 (56.9)	12 (5.8)	1.32	0.52–3.62	0.571

*Significant predictors of non-typhoidal *Salmonella* infection at *p* < 0.05: CI, Confidence interval; Ref, Reference.

### Risk factor analysis for non-typhoidal *Salmonella* infection in slaughterhouse workers

Univariable analysis examining the risk factors associated with NTS in slaughterhouse workers revealed that the odds of NTS positive stool sample (outcome variable) were associated with three predictor variables: geographical location, i.e., eastern region (OR = 2.80, 95% CI: 1.01–8.08, *p* = 0.049), “risky behavior” (considered if the slaughterhouse worker answered ‘yes’ to any of the following: eating, drinking, or smoking) while working (OR = 4.25, 95% CI: 1.38–18.51, *p* = 0.023) and religion (OR = 2.86, 95% CI: 1.07–7.33, *p* = 0.029) (Table 3).

However, region and religion were highly correlated, with only the eastern region having a substantial Muslim population. Pearson chi-square revealed no significant association (*p* = 0.114) between religion and *NTS* infection in the eastern region.

A multivariable model with the above predictor variables revealed that “risky behavior” was significantly associated with NTS positive stool (OR = 1.95, 95% CI: 0.67–2.90%, *p* = 0.004) and eastern region, marginally significant (OR = 1.21 95% CI: 0.62–1.95, *p* = 0.051) (Supplementary Table 3).

## Discussion

In this study, NTS were isolated from 5.2% of the stool samples from livestock slaughterhouse workers, consistent with findings by Kahsay et al. (37). In a systematic review of NTS in Ethiopia between 2010 and 2022, the prevalence of NTS in stool samples from asymptomatic food handlers was between 1 and 10% from a total of 13 studies, and 75% of those studies reported a prevalence of less than 5% (37).

*S*. Typhimurium, which is among the leading causes of gastroenteritis globally was the predominant serovar in the slaughterhouse workers (20). *S*. Typhimurium and *S*. Enteritidis are the main serovars implicated in iNTS in the African region, and are associated with high morbidity and mortality (6, 17, 18, 38–41). Subsequently, it is important to establish whether the *S*. Typhimurium serovars from our study, belong to sequence type (ST) 313, the primary cause of iNTS, through further molecular characterization using next generation sequencing technique (38, 42–45). We did not detect *S*. Enteritidis in the slaughterhouse workers’ population, similar to a study by Afema et al. (25) where they only found two *S*. Typhimurium isolates and no occurrence of *S*. Enteritidis, from archived human clinical isolates. We however identified *S*. Enteritidis in pigs and in different matrices along the pork value chain.

We found a further 10 epidemiologically important serovars in the slaughterhouse workers besides *S*. Typhimurium. *S.* Zanzibar, a zoonotic serovar, has previously been reported as the predominant serovar in pigs in Uganda (30) and also found in pork, flies, water and vegetables at the point of pork retail (46). However, in humans in Uganda, there is no published documentation of *S.* Zanzibar in the studies done so far (25, 27). In addition, both slaughterhouse workers who tested positive for *S.* Zanzibar in this study also reported clinical signs consistent with salmonellosis suggesting that *S.* Zanzibar could be one of the causes of human infection in Uganda. Previously, it has been isolated from humans in the United Kingdom and in Nigeria from children with acute gastroenteritis (47, 48).

*S.* Stanleyville has been identified in clinical isolates from humans in Africa (49, 50). This serovar has been linked to iNTS in West Africa as reported by Tennant et al. (49) and has been associated with a unique case of urinary tract infection in a young boy (51). We found only one isolate of *S.* Stanleyville in the slaughterhouse workers and 4.3% (15/344) in the pork value chain, reiterating the potential of transmission of this important serovar to humans and the public health implications. *S.* Newport is another clinically and epidemiologically important serovar globally. It was our fourth most predominant serovar in the pork value chain. It has been associated with iNTS, multidrug resistance, asymptomatic carriers and is known to survive well in multiple environments and can easily be transmitted to humans (52–55). Previously, Afema et al. (25) detected *S.* subspecies II 42:r:- in humans isolates as well as in ruminants and poultry. In our current study, we additionally identified this serovar in pigs. There is scanty information on *S.* Offa, *S.* Adelaide, *S.* Kenya, *S.* Ituri, *S.* Moroto and *S.* Sanjuan in humans. However, Ndoboli et al. (46) reported *S.* Offa in pork and related fresh-vegetable servings among pork outlets in Kampala, Uganda.

At pig slaughter, we observed NTS prevalence of 19.2% (MLN) and 20.5% (fecal) in slaughtered pigs. Recently, NTS carriage of 12.7% in Busia (Kenya), 9.1% in Nairobi (Kenya) and 24.6% in Chikwawa (Malawi) was established in fecal and MLN of slaughtered pigs (42). Infected pigs are an important source of introduction of NTS into the value chain, increasing the likelihood of carcass contamination (10, 56). Therefore, it is essential to implement NTS control measures on pig farms (10). The prevalence at slaughter we found was higher than the 12% prevalence reported in weaners and piglets at farm level in Uganda (30). Generally, pigs at the farm level show a lower prevalence of *Salmonella* than pigs at the point of slaughter, which could be attributed to latent carriers on the farm and increased *Salmonella* shedding during transport and lairage due to stress (57–59). Key NTS serovars associated with severe gastroenteritis globally were identified in pigs, including Typhimurium, Newport, Enteritidis, Stanleyville, Uganda, Heidelberg and Hadar, which is comparable to the serovars identified by Wilson et al. (42) in Kenya and Malawi.

NTS prevalence observed in pig carcasses (30.5%) was higher compared to the prevalence in their feces (20.5%) and MLN (19.2%), pointing to cross-contamination during the slaughter process (56). Similarly, Kikuvi et al. (60) also found a higher prevalence of 19% NTS in carcass swabs in comparison to 8.6% in fecal samples from pigs slaughtered at Ndumbuini abattoir in Nairobi, Kenya. The high prevalence in floor swabs could be due to the fact that in most of the facilities, we observed no separation of clean and dirty areas; stunning, bleeding, dehairing, evisceration and even carcass splitting was all done on the floor. Water used for dressing carcasses was in buckets and sometimes in jerry cans, increasing the odds of cross-contamination. There was also a likelihood of contaminated meat handlers’ hands contaminating the carcasses (29).

The overall NTS prevalence remained relatively consistent between the point of pig slaughter (23.9%) and pork retail (20.7%), indicating persistence along the chain. The prevalence of NTS in raw pork (33.1%) upon arrival at retail also remained relatively consistent compared to prevalence in carcasses (30.5%) dispatched from slaughter. This contrasts findings from neighboring Kenya, where they observed more than 50% increase in prevalence of NTS in raw pork at retail (28%) compared to NTS prevalence in carcasses (18.1%) (61). Gichuyia et al. (61) attributed this to possible cross-contamination during transport, and in our case additional contamination could be from the retail environment, i.e., chopping surface and hand swabs. The prevalence was notably high in raw pork (33.1%), chopping surfaces (38.1%), and hand swabs (19.6%), but diminishes in cooked pork (4%). This decline can be attributed to thorough cooking, which effectively decreases the bacteria present (61). However, the risk of infection to consumers through contaminated cooked pork remains high, as well as the risk to consumers who buy raw pork to prepare at home (61). There is also a risk of re-introduction of bacteria to cooked pork from raw pork, chopping surfaces, and hands if proper hygiene is not observed (62). Additionally, Ndoboli et al. (46) highlighted the probable risk of vegetables being contaminated by flies. However, in our study, we observed a low prevalence in vegetables, possibly indicating minimal cross-contamination at this point. Overall, the presence of NTS throughout the chain can be attributed to poor hygiene practices and improper pork handling, both during the slaughtering process and at the retail level. Consequently, cross-contamination occurs, elevating the risk of foodborne hazards for both end consumers and workers within the meat industry (23, 62–66). This underscores the need for targeted interventions aimed at enhancing hygiene standards and pork handling practices.

Our study revealed the same serovars persisting along the chain from slaughter to retail for the same pig carcasses, in all the regions, strongly indicating occurrence of cross-contamination. Additionally, we found overlapping serovars in humans, pigs, and within slaughter and retail environments, posing a significant public health concern. Campos et al. (10) showed evidence of serovars from the pork value chain causing infections in humans. Notably, 50% (4/8) of the serovars found in slaughterhouse workers were also found in slaughtered pigs, underscoring the important potential role of pigs as reservoirs for NTS. Wilson et al. (42) recently confirmed that pigs in Kenya and Malawi serve as reservoirs for a diverse range of zoonotic NTS serovars. Additionally, we identified several zoonotic serovars along the value chain including, Agona, Heidelberg, Hadar, Montevideo, Braenderup (11). It is imperative to conduct phylogenetic analyses of these serovars to ascertain relatedness and trace their sources using sequencing techniques. Afema et al. (25) reported shared NTS serovars, genotypes, and AMR phenotypes among isolates from humans, animals, and the environment, highlighting the potential role of animal source foods and the environment as important reservoirs for NTS in Uganda.

Globally, *S*. Typhimurium has increasingly developed resistance, particularly in the African region due to its association with iNTS infections, that often require antibiotic treatment (17, 18, 38, 40). Specifically, resistance to tetracyclines and sulfonamides has been widely reported both in humans and pigs, with tetracycline resistance in pigs linked to its use in the production chain (67). Our study revealed phenotypic multidrug resistant *S*. Typhimurium isolates from humans to tetracycline, sulfamethoxazole and tigecycline, specifically from Mbale district in the eastern region, and this needs to be further elucidated. Contrary to findings by Bosco et al. (26) where resistance to chloramphenicol was the most prevalent, our findings point to a new trend of resistance. Notably, all the three commonly used antibiotics in the treatment of salmonellosis; chloramphenicol, nalidixic acid and ciprofloxacin were all sensitive. The resistance observed in humans therefore may indicate antibiotics commonly used in animals, as previous studies show evidence of transmission of resistance from animals to humans, specifically for *Salmonella* (10, 26). There is evidence that NTS from pork and pork products has high levels of antimicrobial resistance (45). It was however not within the scope of the current study to test the animal isolates for antimicrobial resistance which should be the focus of future work.

The majority of the slaughterhouse workers with NTS (52.6%) did not exhibit symptoms typically associated with NTS, such as diarrhea, vomiting, headache, fever, nausea, and abdominal pain. This could be attributed to asymptomatic individuals as demonstrated by Kariuki et al. (40) who showed evidence of asymptomatic human carriers in Kenya. Falay et al. (44) also established that healthy human carriers are potential reservoirs for iNTS, specifically *S*. Typhimurium ST313.

The univariable analysis showed that slaughterhouse workers from the eastern region had a higher likelihood of NTS-positive stool samples compared to the northern region. This needs to be further investigated, as there were no plausible explanations in our study, and no other studies were available for comparison with our findings. Additionally, we observed that all *S*. Typhimurium-positive stool samples were from a single ruminant slaughterhouse in Mbale, eastern region and exhibited the same antimicrobial resistance profiles except one isolate. This suggests a possible NTS outbreak, with the same serovar circulating among the slaughterhouse workers. This may also explain the observed relationship between religion and positive stool samples, as religion and region were highly correlated, with the eastern region having a substantial Muslim population. The potential for outbreaks in this occupational setting should be further investigated and control measures implemented particularly if there are ongoing veterinary public health issues in this region.

Our findings revealed poor hygiene and hand washing practices at slaughter. Notably, none of the slaughterhouse workers reported using hand gloves as part of their personal protective wear, indicating direct contact with contaminated animal feces, carcasses, and environment. Consequently, individuals engaging in risky behaviors such as eating, drinking, or smoking while working are at a significantly higher risk of *Salmonella* carriage (68). Ingestion of *Salmonella* during work may explain the positive association with the presence of NTS. We also observed that the level of training was generally very low (9.6%). Research has established that training leads to notable improvements in knowledge, hygiene and meat handling practices, resulting in safer products and reduced occupational risks (69). There is need for targeted training programs, focusing on hygiene and behavioral practices among slaughterhouse workers.

### Study limitations

The occurrence of NTS serovars in cattle and small ruminants was not explored due to time and budget constraints. We did not quantify *Salmonella* in cooked pork and vegetables, a crucial aspect of quantitative microbial risk assessment that evaluates the potential to cause infection in humans (66). Future studies should therefore consider slaughter and retail points of cattle, sheep, and goats. They should also quantify the risk of salmonellosis in humans, conduct antimicrobial susceptibility testing for pork and environmental isolates, and perform phylogenetic analyses of the isolated NTS serovars.

## Conclusion

The current study provides significant insights into prevalence, circulating serovars, and antimicrobial resistance patterns of NTS infection in slaughterhouse workers in Uganda, as well as in the pork value chain. This also contributes to the limited data available on the burden of foodborne diseases in low- and middle-income countries. The serovars identified in humans are a potential indicator of the circulating serovars and potential cause of disease within the general human population, highlighting the importance of using slaughterhouses as sentinels for zoonotic disease surveillance. The antimicrobial resistance observed in human isolates also provide insight into the potential limitations of antimicrobials used in both animal production and human context. Our study additionally demonstrates the potential risk of transmitting NTS to meat consumers through contaminated meat, underscoring the need to enhance hygiene and meat handling practices at the point of slaughter and retail.

## Data Availability

The original contributions presented in the study are included in the article/Supplementary material, further inquiries can be directed to the corresponding authors.
