# Supplementary material for: Non-typhoidal Salmonella among slaughterhouse workers and in the pork value chain in selected districts of Uganda
Source: Front Vet Sci. 2024 Sep 17;11:1427773. doi: 10.3389/fvets.2024.1427773 (PMC11472856; doi:10.3389/fvets.2024.1427773)
Supplement: Supplementary file 3 [file Table_3.DOCX]

S2 Table: Multivariable analysis for infection with non-typhoidal *Salmonella* in slaughterhouse workers

|  | Non-typhoidal *Salmonella* | | |
| --- | --- | --- | --- |
| Predictors | Odds ratio | 95% CI | p value |
| Region 1- northern | 1 | Reference | Reference |
| Region 2-eastern | 1.21 | 0.62-1.95 | **0.051** |
| Religion 2- Muslim | 0.67 | 0.56-1.19 | 0.236 |
| Risky behavior | 1.95 | 0.67-2.90 | **0.004** |
