# Supplementary material for: Non-typhoidal Salmonella among slaughterhouse workers and in the pork value chain in selected districts of Uganda
Source: Front Vet Sci. 2024 Sep 17;11:1427773. doi: 10.3389/fvets.2024.1427773 (PMC11472856; doi:10.3389/fvets.2024.1427773)
Supplement: Supplementary file 4 [file Data_Sheet_1.PDF]

MAKERERE UNIVERSITY SCHOOL OF HEALTH SCIENCES RESEARCH AND ETHICS COMMITTEE (MAKSHS-REC)

INFORMED CONSENT FORM FOR THE STUDY ON THE EPIDEMIOLOGY OF ZOONOSES AMONG SLAUGHTERHOUSE WORKERS IN UGANDA FOR RESEARCH PARTICIPANTS AGED 18 YEARS AND ABOVE

Title of the proposed study:

EPIDEMIOLOGY OF ZOONOSES AMONG SLAUGHTERHOUSE WORKERS IN UGANDA

Investigators:

1. James Bugeza: Makerere University, Uganda, and International Livestock Research Institute (ILRI)
2. Lordrick Alinaitwe: University of Bern, Switzerland and ILRI
3. Velma Kivali: Free University of Berlin, Germany and ILRI
4. Elizabeth Cook: ILRI
5. Kristina Roesel: ILRI
6. Jolly Justine Hoona: Ministry of Agriculture Animal Industries and Fisheries (MAAIF) Uganda

Study sponsor

This research is funded by the German Federal Ministry for Economic Cooperation and Development (BMZ), through ILRI in collaboration with the Uganda Ministry of Agriculture, Animal Industry and Fisheries (MAAIF). Elizabeth and Jolly are the project coordinators while Lordrick, Velma and James are research fellows directly recruited to design and implement studies aimed at investigating the epidemiology of select zoonoses among slaughter workers.

Background and rationale for the study:

The meat value chain in Uganda has been reported to present serious food borne and occupational safety risks to both consumers and meat handlers at various levels, with animal slaughter being identified as the critical control point. Slaughter animals in Uganda have unknown disease history and are sourced from various geographical locations within and across the borders of Uganda. Abattoir workers in Uganda slaughter various types of food animals, mainly cattle, goats, sheep, pigs and poultry.

Of recent, several diseases including brucellosis, leptospirosis and salmonellosis have been found in food animals in Uganda. The above diseases can spread from animals to humans, and those in direct contact with animals including slaughterhouse workers are at the highest risk.

The aim of the current research is to derive information on the occurrence of these diseases in both the slaughtered animals and workers, and circumstances under which the causative pathogens spread. Such information will help inform measures for prevention and control of these diseases in both humans and animals in Uganda.

Purpose:

To understand circumstances under which *Brucella*, *Leptospira* and *Salmonella* pathogens spread between humans and animals, and use such information to inform measures for prevention and control of these pathogens in both humans and animals in Uganda.

Procedures:

If you decide to participate, we will take 20-30 minutes asking you questions majorly relating to your work at the slaughterhouse. We will also ask personal questions including your age, sex, contact information and request to take a blood sample and/or stool sample from you. The blood sample will be collected by a trained nurse while we shall provide the necessary materials and a private room to enable participants collect a stool sample.

Who will participate in the study and where the study is going to be conducted from?

This study targets meat handlers from five geographic regions of Uganda, who are directly involved in butchering, processing, and retail of ruminant meat and pork. The specific areas are Kampala in the Central, Arua in West Nile, Lira/Gulu in North, Mbarara in the Western/Southern and Mbale/Soroti in the East.

Risks/Discomforts:

There are no serious foreseeable risks associated with participation in this study. None-the-less a few participants may experience slight temporary discomfort during blood sampling. Our field medical team will offer necessary attention to such participants.

Benefits to the participants:

A free medical checkup will be offered upon which you will be given advice or appropriate referrals. As part of the routine requirement for food handlers, you will instantly know your status regarding Brucella exposure; while results for Leptospira and Salmonella shall be relayed later. The longer-term benefits of the study will be creation of awareness of zoonotic diseases and the formulation of policy guidelines relating to the prevention and control of zoonoses.

Cost:

There will be no costs incurred by the participants of this study.

Compensation for participation in the study:

If you decide to participate, you will be compensated with UGX 10,000 equivalent to the time we anticipate you will lose in the interview and sample collection. In the most unlikely event that you suffer any injury during sample collection, our field medical personnel will offer the necessary treatment and support. The extent of invasiveness associated with blood collection techniques used in this study do not cause serious medical injury that could be feared to cause permanent damage.

Reimbursement:

We intend to enrol all participants at their respective work areas. However, should there be need for one to travel to a place beyond their work area for the purpose of this study, the project will reimburse associated travel costs.

Questions about the study:

If you later have any further questions relating to this study, please do not hesitate to contact the following research team members: James Bugeza, Email- J.bugeza@cgiar.org , (+256772523516); Lordrick Alinaitwe, Email- L.alinaitwe@cgiar.org (+256775460777); Velma Kivali , Email- V.kivali@cgiar.org (+254720553993) and Elizabeth Annie Cook , Email E.cook@cgair.org (+254713097560).

Questions about participants rights:

Participants who have questions regarding their welfare and rights as research participants can have their questions addressed by the Chairperson, Makerere University School of Health Science IRB (MakSHSIRB) Dr. Kalidi Rajab on telephone number +256 776798978 or +256 0200903786)

Research involving the collection of human materials/samples

Collected samples will be used for analysis of *Brucella*, *Leptospira* and *Salmonella* pathogens to directly address objectives of this study. The remaining samples will be stored for future use. The samples will remain with unique anonymous identifiers at all time; and once all analysis is complete, the remaining material may be destroyed after consultation with Uganda National Council for Science and Technology.

Dissemination of study feedback or study findings and progress of the study

All results from this survey may not be returned to you but will be compiled into a final report for submission to health professionals and/or government officials. This may include suggestions for preventive measures to reduce the risk of getting these diseases.

Statement of voluntariness:

We would like to emphasise that your participation is voluntary, and you are free to withdraw at any point.

Ethical approval of the research study

This study has been approved by Makerere University School of Health Sciences Research and Ethics Committee /IRB) which is an accredited Ugandan based Research and Ethics Committee/IRB.

Confidentiality

All information collected shall be kept anonymous, and as guided by international and local ethical standards governing research involving humans as research participants. My identity will be concealed, and my name will not appear anywhere on the coded forms with the information. The study team will be the only one with the authority to access the collected data. However, the School of Health Sciences Research and Ethics Committee and the Uganda National Council for Science and Technology (UNSCT) may have access to private information that identifies the research participants by name where applicable. The filled questionnaire or any other filled data collection form will be kept under strict lock and key, and information on computers will be kept confidential with password protection respectively. For any further questions, I may contact the Chairperson of the School of Health Sciences Research and Ethics Committee (MakSHSREC) on (+256) +256 776798978 / (+256) 0200903786 or Uganda National Council of Sciences and Technology on Tel: (+256)-041-4705500).

STATEMENT OF CONSENT

.... has described to me what is going to be done, the risks, the benefits involved and my rights regarding this study. I have been informed about the study in which I am voluntarily agreeing to take part. In the use of this information, my identity will be concealed. I am aware that I may withdraw at any time. I understand that by signing this form, I do not waive any of my legal rights but merely indicate that I have been informed about the research study in which I am voluntarily agreeing to participate. A copy of this form will be provided to me.

Name of research participant…………………………………………………………………….

Age…………………………………………………………………………………………….

Signature/thumbprint ………………………………………………………………………….

Date (DD/MM/YY)……………………………………………………………………………….

Name of Witness ……………………………………………………………………………………. .

Signature ………………………………………………………………………………………...

Date (DD/MM/YY)……………………………………………………………………………...

Name of the person consenting the research participant:……………………………………….

Signature ………………………………………………… …………………………………….

Date (DD/MM/YY)………………………...
