# Supplementary material for: Non-typhoidal Salmonella among slaughterhouse workers and in the pork value chain in selected districts of Uganda
Source: Front Vet Sci. 2024 Sep 17;11:1427773. doi: 10.3389/fvets.2024.1427773 (PMC11472856; doi:10.3389/fvets.2024.1427773)
Supplement: Supplementary file 5 [file Data_Sheet_2.PDF]

QUESTIONNAIRE FOR SLAUGHTERHOUSE WORKERS

Date ……………… **District**………………………… **Questionnaire No..**……………

Enumerator ……………………**Slaughter facility name** ……………………………...

Sample collected: ☐ Whole blood ☐ Serum ☐ Stool

Part 1: DEMOGRAPHIC FACTORS

1.1 Age………years

1.2 Sex ☐ Female ☐ Male

1.3 What religion are you?

☐ Christian ☐ Muslim ☐ SDA ☐ Other (specify)………………….

1.4 Which level of education did you stop at?
☐ No formal education ☐ Primary level ☐ Secondary level ☐ Tertiary level

1.5 Have you attended any training related to animal slaughter and meat handling in the last 6 months?
☐ Yes ☐ No

1.6 Did the training above cover any of the following topics/themes?
☐ Meat hygiene ☐ Zoonotic diseases ☐ Occupational safety ☐ Personal hygiene

Part 2: WORK RELATED RISK FACTORS

2.1 When did you last enter the slaughter facility?................................ days ago

2.2 What do you currently do in this slaughter facility?

☐ Slaying (Halal butcher)	☐ Limb cutting	☐ Dealer in horns
☐ Blood collection	☐ Meat cutting and loading	☐ Dealer in dog feed parts
☐ Skinning/ Dehairing	☐ Meat flake picking	☐ Live animal trader
☐ Evisceration	☐ Dealer in heads	☐ Offloading animals
☐ Decapitation	☐ Dealer in limbs	☐ Transporting live animals
☐ Upholstering	☐ Dealer in offals	☐ Transporting meat
☐ Washing offal	☐ Dealers in offcuts	☐ Leading animal to slaughter line
☐ Upholstering	☐ Dealer in kidneys	☐ Cleaning the facility
☐ Washing offal	☐ Dealer in plucks (lung, liver, heart, spleen)	☐ Administration/Management
☐ Carcass splitter	☐ Dealer in testis/udder/penis	Others………………………………… …………………………………………
☐ Meat inspection	☐ Dealer in skins	

2.3 How long have you carried out the activities you have mentioned above?................. months

2.4 Which animals do you currently handle the most at this abattoir?
☐ Cattle ☐ Goats & sheep ☐ Pigs

2.5 How many days do you usually work in a month?
☐ Daily ☐ A few days a week ☐ Entire month

2.6 On a particular day that you work, how many animals/ carcasses/ parts do you handle?
☐ 1-5 ☐ 6-10 ☐11-20 ☐ 21-50 ☐ Over 50 ☐ Not applicable

2.7 On a particular day that you work, how many hours do you have to work?
☐ Less than an hour ☐ 1-2 hours ☐ 3-5 hours ☐ 6-10 hours

2.8 How often do you find yourself in a situation where you have to drink or eat while working?
☐ Daily ☐ Atleast once a month ☐ Can`t remember the last time ☐ Never

2.9 How often do you find yourself in a situation where you have to smoke while working?
☐ Daily ☐ Atleast once a month ☐ Can`t remember the last time ☐ I don`t smoke

2.10 How often do you wear the following attire while working?

	All the time	Sometimes	Never
Gumboots			
Overalls/aprons			
Safety googles/face shield			
Gloves			
Hair cover			

2.11 How often do you clean your apron / overall?
☐ Daily ☐ Atleast once a week ☐ At least once or twice a month ☐ After over a month
☐ Whenever I deem it dirty

2.12 How often do you clean your gumboots?
☐ Daily ☐ Atleast once a week ☐ At least once or twice a month ☐ After over a month
☐ Whenever I deem it dirty

2.13 Have you experienced any of the following accidental events in the last three months?

	**YES**	**NO**
Animal blood splash in your eyes		
Animal urine splash in your eyes		
Cut on your hands or legs		

2.14 When did you last work with an open wound?
☐ Less than a week ago ☐ 1–4 weeks ago ☐ 5-50 weeks ago
☐ One year ago ☐ I have Never had a wound

2.15 While at work , when do you find yourself in a situation where you have to wash your hands?

☐ Before handling carcass/meat/animal parts
☐ After handling carcasses /meat/animal parts
☐ Before eating ☐ After visiting the toilet ☐ Following an injury

2.16 Kindly describe how you usually wash your hands
☐ Water only ☐ Water and soap ☐ Use disinfectant ☐ Never wash hands

Part 3: ACTIVITY OUTSIDE SLAUGHTERHOUSE

3.11 What kind of animals do you have at your home?

☐ Dogs ☐ Cattle ☐ Goats ☐ Sheep ☐ Pigs ☐ Rabbits
☐ Donkey/Oxen/Horse ☐ Fish ☐ Chicken ☐ I don`t keep animals at all
☐ Others:................ ....

3.12 How often do you have to participate in feeding those animals you mentioned above?

	Daily	Atleast once a week	May not in a week but not for a month	A few times over the year	Not in the last one year
Dogs					
Cattle					
Goats					
Sheep					
Pigs					
Donkey/Oxen/Horse					
Fish					
Chicken					
Others:..					

3.13 How often do you have to participate in cleaning for those animals you mentioned above?

	Daily	A few times a Week	May not in a week but not for a month	A few times a year	Not in the last one year
Dogs					
Cattle					
Goats					
Sheep					
Pigs					
Donkey/Oxen/Horse					
Fish					

Chicken					
Others:..					

3.14 How often do those animals you mentioned above enter your house?

	Daily	A few times a Week	May not in a week but not for a month	A few times a year	Not in the last one year
Dogs					
Cattle					
Goats					
Sheep					
Pigs					
Donkey/Oxen/Horse					
Fish					
Chicken					
Others:...					

3.15 How often do you get involved in the following activities?

	Daily	A few times a week	May not in a week but not for a month	A few times a year	Not in the last one year
Swimming in rivers, lake, pond/stream					
Fishing					
Handling abortions					
Rice/sugarcane farming					
Milking					
Assisting with animal births					
General gardening/digging					

3.5 How often do you see rats or mice near your house?

☐ Daily ☐ At least once a week ☐ May not in a week but not for a month
☐ Can`t take a year without ☐ Never see

3.6 How often do you see signs of rodent dropping or feeding traces in the vicinity of your house?

☐ Daily ☐ At least once a week ☐ May not in a week but not for a month
☐ Can`t take a year without ☐ Never see

3.7 What is the source of water you use at your home?
☐Tap/pipped water ☐ Borehole ☐ Open wells ☐ Communal reservoir/dam
☐ Spring ☐ River/stream ☐ Lake ☐ Pond ☐ Tank

3.8 Have you felt unwell in the last one month? ☐ Yes ☐ No

3.9 If yes, what symptoms did you have?

☐ Vomiting	☐ Headache	☐ Muscle pain	☐ Red urine
☐ Night sweats	☐ Fatigue	☐ Weight loss	☐ Lack of appetite
☐ Diarrhoea	☐ Yellowing of eyes	☐ Joint pain	☐ Fever
☐ Abdominal pain	☐ Nausea		
Others:			

3.10 Did you still go to work then?
☐ Yes ☐ No

4.1 Do you have any known health conditions that require you making regular visits to hospital?
☐ Yes ☐ No

4.2 You may share if only you do not mind to: ………………………………………………...

Thank you for your participation in this study!
